# Development of a clinical and bronchoalveolar lavage fluid biomarker model for predicting post-infectious bronchiolitis obliterans in hospitalized children

**DOI:** 10.3389/fped.2026.1889551

**Published:** 2026-10-13

**Authors:** Guobin Cui, Haibin Lin, Fenghua Cheng, Saiping Huang

**Affiliations:** 1 Department of Pediatric Intensive Care Medicine, The Affiliated Hospital of Putian University, Putian, China; 2 Department of Ultrasound, The Affiliated Hospital of Putian University, Putian, China; 3 Department of Pediatric Respiratory Medicine, The Affiliated Hospital of Putian University, Putian, China

**Keywords:** bronchoalveolar lavage fluid, children, inflammatory biomarkers, post-infectious bronchiolitis obliterans, prediction model, nomogram

## Abstract

**Objective:**

To evaluate previously reported clinical factors and explore whether acute-phase bronchoalveolar lavage fluid (BALF) inflammatory biomarkers measured during hospitalization provide additional prognostic information for subsequent post-infectious bronchiolitis obliterans (PIBO).

**Methods:**

This single-center retrospective cohort included 220 hospitalized children with lower respiratory tract infection who underwent bronchoscopy and BALF testing for clinical indications. BALF biomarkers were measured during the index hospitalization, before PIBO ascertainment at follow-up approximately 2 months after infection. Patients were randomly divided into a model-development cohort (*n* = 155) and an internal holdout cohort (*n* = 65). Logistic regression was used to construct clinical, BALF biomarker, and combined models. Model development followed the TRIPOD + AI framework, with evaluation of discrimination, calibration, clinical net benefit, bootstrap optimism, and penalized-regression sensitivity analyses.

**Results:**

During follow-up, 49/220 children (22.3%) met PIBO diagnostic criteria. In the training cohort, PIBO was associated with adenovirus or mixed infection, longer fever duration, PICU admission, mechanical ventilation, and higher BALF matrix metalloproteinase-9, interleukin-8, and myeloperoxidase (MPO) levels (all *P* < 0.001). In multivariable analysis, each 1-SD increase in fever duration (*OR* = 1.72, *95% CI*, 1.03–3.01; *P* = 0.045) and BALF MPO (*OR* = 1.93, *95% CI*, 1.13–3.61; *P* = 0.028), adenovirus infection (*OR* = 4.68, *95% CI*, 1.36–20.03; *P* = 0.021), and mechanical ventilation (*OR* = 4.04, *95% CI*, 1.30–13.08; *P* = 0.016) remained associated with PIBO. Clinical, BALF biomarker, and combined models yielded AUCs of 0.846, 0.815, and 0.860 in training and 0.718, 0.703, and 0.723 in the holdout cohort. Bootstrap-corrected AUC for the combined model was 0.822. In ridge sensitivity analysis, holdout AUCs were 0.742 for the combined model and 0.706 for the clinical model (DeLong *P* = 0.475).

**Conclusion:**

Adenovirus infection, prolonged fever, mechanical ventilation, and higher acute-phase BALF MPO were associated with subsequent PIBO. However, adding BALF biomarkers produced only small, non-significant improvements in holdout discrimination. These findings are exploratory and require prospective external validation in larger multicenter cohorts before clinical application.

## 1 Introduction

Lower respiratory tract infections are common pediatric diseases. After acute infection, some children may develop persistent cough, wheezing, dyspnea, and reduced exercise tolerance; in severe cases, these conditions may progress to post-infectious bronchiolitis obliterans (PIBO) (1). PIBO is a chronic pulmonary disease characterized by chronic small-airway inflammation, fibrosis, and irreversible airflow limitation, and it frequently occurs after severe pneumonia or adenovirus infection (2, 3). Because the early clinical manifestations of PIBO are nonspecific, they may be easily confused with post-infectious cough, wheezing disorders, or bronchial asthma. Consequently, some children may already have evident small-airway injury and impaired pulmonary function at the time of diagnosis (4). Therefore, early identification of children at high risk of PIBO after lower respiratory tract infection is important for improving follow-up management and prognostic assessment.

At present, there is no single specific diagnostic biomarker for PIBO. Clinical diagnosis mainly relies on a history of severe lower respiratory tract infection, persistent respiratory symptoms, chest high-resolution computed tomography findings suggestive of small-airway disease, such as mosaic perfusion or air trapping, and fixed airflow limitation shown by pulmonary function tests (5). However, radiological abnormalities are usually more evident after disease progression, and pulmonary function testing in children is limited by age and cooperation, making early identification challenging (6). Previous studies have suggested that adenovirus infection, severe pneumonia, mechanical ventilation, persistent fever, and enhanced inflammatory responses may be associated with the development of PIBO. Nevertheless, risk assessment based solely on clinical indicators remains limited (7). Therefore, identifying objective indicators that reflect local airway inflammation and combining them with clinical characteristics may help improve the early identification of PIBO.

Bronchoalveolar lavage fluid (BALF) may more directly reflect the local inflammatory microenvironment of the lower respiratory tract (8, 9). MMP-9, IL-8, and MPO have been associated with biological processes related to extracellular matrix turnover, neutrophil recruitment and activation, and oxidative inflammatory activity, respectively. Their concentrations in BALF may therefore reflect the local inflammatory state during severe lower respiratory tract infection, although their specific roles in the development of PIBO remain uncertain. Compared with peripheral blood inflammatory indicators, BALF inflammatory biomarkers may better reflect lesion-site inflammation and may have potential value in assisting the assessment of PIBO risk (10).

Based on these considerations, this study included hospitalized children with lower respiratory tract infection who underwent bronchoscopy and BALF testing. We first examined whether previously reported clinical factors, including adenovirus infection, prolonged fever, and mechanical ventilation, were also associated with subsequent PIBO in this selected cohort. The principal exploratory objective was to evaluate whether acute-phase BALF levels of MMP-9, IL-8, and MPO measured during hospitalization were associated with subsequent PIBO and whether these biomarkers provided additional prognostic information beyond routinely available clinical variables. Because the biomarkers were measured during the acute infectious episode, they were considered candidate prognostic markers rather than PIBO-specific diagnostic biomarkers.

## 2 Study population and methods

### 2.1 Study population

This was a single-center retrospective cohort study that included pediatric patients with lower respiratory tract infection admitted to The Affiliated Hospital of Putian University between June 2023 and May 2025. All children underwent bronchoscopy for clinical diagnostic or therapeutic purposes, during which bronchoalveolar lavage fluid (BALF) samples were collected. According to whether PIBO was diagnosed at the follow-up assessment, generally performed approximately 2 months after the index infection, the patients were classified into the PIBO and non-PIBO groups.

The inclusion criteria were as follows: (1) age ≤18 years; (2) diagnosis of pneumonia, bronchopneumonia, or other lower respiratory tract infections; (3) completion of bronchoscopy and BALF testing during hospitalization; and (4) availability of complete clinical and follow-up data. The exclusion criteria were as follows: (1) previous confirmed diagnosis of PIBO, bronchiectasis, or other chronic small-airway diseases; (2) presence of severe congenital airway malformation, immunodeficiency, or other underlying diseases that could affect outcome assessment; (3) missing data on BALF inflammatory biomarkers or primary outcomes; and (4) incomplete follow-up data.

A total of 220 children were included in this study, including 155 in the training cohort and 65 in the validation cohort. During follow-up, 49 of the 220 included children met the diagnostic criteria for PIBO. Because bronchoscopy and BALF collection were performed for clinical indications rather than routine or population-based screening, the included patients represented a selected, higher-risk subgroup of hospitalized children with more severe, persistent, or diagnostically challenging lower respiratory tract infections. Therefore, the observed proportion of PIBO in this cohort should not be interpreted as a population-based prevalence estimate, and the model should be considered primarily applicable to similar hospital-based populations. The training cohort was used for model development, and the validation cohort was used for model performance evaluation.

This study was approved by the Medical Ethics Committee of The Affiliated Hospital of Putian University (approval No. PYFL[2025176]). Given the retrospective nature of the study and the anonymization of all patient data, the requirement for informed consent was waived by the ethics committee.

### 2.2 Data collection

Demographic information, clinical data, laboratory findings, pathogen results, and treatment-related data were collected through the electronic medical record system. Demographic information included age, sex, body weight, history of premature birth, underlying diseases, and previous wheezing history. Clinical data included duration of fever, length of hospital stay, oxygen therapy, pediatric intensive care unit (PICU) admission, and mechanical ventilation. Pathogen data included adenovirus, Mycoplasma pneumoniae, respiratory syncytial virus, mixed infection, and unidentified pathogens.

Laboratory indicators included white blood cell count, neutrophil percentage, C-reactive protein, procalcitonin, and lactate dehydrogenase. BALF-related indicators included BALF neutrophil percentage and the levels of MMP-9, IL-8, and MPO.

### 2.3 Bronchoscopy, BALF collection, and biomarker testing

Bronchoscopy was not routinely performed at the onset or during a predefined early phase of the infectious episode and was not scheduled according to a protocol-defined research time point. In routine clinical practice, it was generally considered when chest imaging demonstrated marked or persistent pulmonary consolidation, particularly when the clinical or radiological response to initial treatment was insufficient, or when lower-airway pathogen sampling or therapeutic lavage was clinically required. Persistent consolidation, infiltrates, and atelectasis have also been reported as common indications for pediatric flexible bronchoscopy (11, 12).

The decision to perform bronchoscopy was made individually by the treating team after assessment of the evolution of the imaging abnormalities, treatment response, anticipated diagnostic or therapeutic benefit, and procedural risk. Consequently, the timing of bronchoscopy differed among patients and could have represented different stages of the acute inflammatory process. Exact patient-level intervals from infection onset or hospital admission to bronchoscopy could not be reliably reconstructed from the archived records.

Bronchoalveolar lavage was performed in the lung segment corresponding to the predominant radiological lesion, and BALF samples were collected during the procedure and promptly submitted for laboratory testing. BALF concentrations of MMP-9, IL-8, and MPO were measured using commercially available Quantikine enzyme-linked immunosorbent assay (ELISA) kits (R&D Systems, Minneapolis, MN, USA), including the Human MMP-9 Quantikine ELISA Kit (catalog no. DMP900), Human IL-8/CXCL8 Quantikine ELISA Kit (catalog no. D8000C), and Human Myeloperoxidase/MPO Quantikine ELISA Kit (catalog no. DMYE00B), in accordance with the manufacturer's instructions. Because of the retrospective design, complete historical information regarding assay lot numbers, replicate measurements, assay-specific quality-control parameters, and formal laboratory blinding to subsequent PIBO outcome status could not be reliably reconstructed from the archived records. BALF samples were collected during the index hospitalization, before PIBO outcome ascertainment. Accordingly, BALF MMP-9, IL-8, and MPO were evaluated as baseline candidate prognostic biomarkers for subsequent PIBO rather than as contemporaneous diagnostic biomarkers. Because bronchoscopy was performed according to clinical indications rather than at a uniform protocol-defined time point, BALF samples may have represented different stages of the acute inflammatory response. In addition, treatment before BALF collection, including potential exposure to antibiotics, systemic corticosteroids, antiviral agents, or other anti-inflammatory therapies, was not standardized according to a research protocol. The timing and intensity of these treatments could not be comprehensively incorporated into the present analysis. Consequently, biomarker concentrations may have been influenced by disease severity, bronchoscopy timing, and treatment exposure in addition to the underlying airway inflammatory state.

### 2.4 Outcome definition and ascertainment

The primary outcome was PIBO diagnosed at the follow-up assessment, which was generally performed approximately 2 months after the index lower respiratory tract infection. PIBO was defined by: (1) persistent or recurrent respiratory symptoms after the acute infectious episode, including cough, wheezing, dyspnea, exercise intolerance, or persistent hypoxemia; (2) follow-up HRCT findings compatible with small-airway disease, including mosaic attenuation, air trapping, bronchial wall thickening, or bronchiectasis; and (3) specialist assessment after exclusion of alternative diagnoses, including asthma, congenital airway abnormalities, cystic fibrosis, primary ciliary dyskinesia, immunodeficiency-related lung disease, and pre-existing bronchiectasis.

Pulmonary function testing was considered supportive when technically feasible. Evidence of persistent obstructive ventilatory impairment or air trapping supported the diagnosis but was not mandatory because reliable pulmonary function testing could not be completed in some younger children. All suspected PIBO cases were evaluated by pediatric respiratory specialists on the basis of the clinical course, follow-up imaging findings, available pulmonary function results, and exclusion of competing diagnoses.

Follow-up HRCT findings used for PIBO outcome ascertainment were not included as predictors to avoid post-baseline information and outcome leakage. The exact patient-level dates on which the diagnostic criteria were first fulfilled could not be reliably retrieved because of the retrospective design and limitations of the archived information system. Therefore, the mean or median interval from the index infection to PIBO diagnosis could not be calculated.

Outcome status was assigned on the basis of the documented follow-up assessment by pediatric respiratory specialists. Children were classified as having PIBO only when persistent or recurrent respiratory manifestations, compatible follow-up HRCT findings, and exclusion of relevant alternative diagnoses were documented. Pulmonary function findings were used as supportive evidence when technically feasible but were not required for outcome classification.

### 2.5 Model development and evaluation

Three prediction models were evaluated. The clinical model included age, fever duration, adenovirus infection, PICU admission, and mechanical ventilation. The BALF biomarker model included BALF MMP-9, IL-8, and MPO levels. The combined model included all five clinical predictors and the three BALF biomarkers. Candidate predictors were selected on the basis of previously reported associations with PIBO, clinical relevance, availability during the index hospitalization, and the study objective of evaluating whether BALF biomarkers provided additional prognostic information beyond routinely available clinical factors. Univariable *P* values were used to describe crude associations but were not used as the sole criterion for predictor selection.

The training cohort contained 35 PIBO events. The clinical, BALF biomarker, and combined models contained 5, 3, and 8 predictor parameters, corresponding to events-per-variable values of 7.0, 11.7, and 4.4, respectively. The relatively low EPV of the combined model indicated a risk of coefficient instability and overfitting.

Conventional multivariable logistic regression was used to estimate adjusted associations and construct the graphical nomogram. The conventional combined logistic regression model was specified as follows:

$$\begin{array}{rl} \mathrm{LP}= & -3.055+0.232\times \mathrm{Age}\_z+0.545\times \mathrm{Fever}\;\mathrm{duration}\_z+1.542\\ \times & \mathrm{Adenovirus}\;\mathrm{infection}+1.397\times \mathrm{Mechanical}\;\mathrm{ventilation}-0.064\\ \times & \mathrm{PICU}\;\mathrm{admission}+0.404\times \mathrm{BALF}\;\mathrm{MMP}-9\_z+0.303\\ \times & \mathrm{BALF}\;\mathrm{IL}-8\_z+0.659\times \mathrm{BALF}\;\mathrm{MPO}\_z. \end{array}$$

The predicted probability of PIBO during follow-up was calculated as:

P(PIBO)=exp(LP)/[1+exp(LP)].

Adenovirus infection, mechanical ventilation, and PICU admission were coded as 1 for presence and 0 for absence. Continuous predictors were standardized as z = (observed value−training-cohort mean)/training-cohort standard deviation. The training-cohort means and standard deviations were 33.94 and 17.67 months for age, 8.59 and 3.13 days for fever duration, 318.00 and 125.67 ng/mL for BALF MMP-9, 319.43 and 150.22 pg/mL for BALF IL-8, and 249.69 and 120.76 ng/mL for BALF MPO, respectively.

Continuous predictors were standardized using the means and standard deviations of the training cohort. Model discrimination was assessed using receiver operating characteristic curves and the area under the curve. Calibration was evaluated using calibration curves and the Hosmer–Lemeshow test.

To quantify model optimism, internal validation of the complete combined model was performed using 1,000 bootstrap resamples. In each bootstrap sample, the model was refitted and evaluated in both the bootstrap sample and the original training cohort. Optimism-corrected AUC, calibration intercept, calibration slope, and Brier score were calculated. At the cutoff derived from the training cohort, the positive likelihood ratio (LR+) was calculated as sensitivity/(1−specificity), and the negative likelihood ratio (LR−) was calculated as (1−sensitivity)/specificity.

The incremental predictive contribution of adding BALF biomarkers to the clinical model was additionally explored in the internal holdout cohort using continuous net reclassification improvement and integrated discrimination improvement. Because no clinically established risk categories were prespecified, categorical NRI was not calculated. Confidence intervals for continuous NRI and IDI were estimated using 5,000 stratified bootstrap resamples.

The nomogram was constructed from the full conventional combined logistic regression model and used exactly the same eight prespecified predictors, coding scheme, standardization procedure, and regression coefficients as that model. Predictors were retained regardless of their individual multivariable *P values* because predictor inclusion was based on the prespecified model structure rather than data-driven significance screening. Ridge-penalized regression was conducted only as a sensitivity analysis and was not used to construct the nomogram.

Ridge-penalized logistic regression was additionally performed as a sensitivity analysis to reduce coefficient instability. The penalty parameter was selected using stratified 10-fold cross-validation, and the more conservative lambda.1se value was applied. The 65-patient validation cohort was considered an internal holdout cohort rather than an external validation cohort.

### 2.6 Statistical analysis

Statistical analyses were performed using R software (version 4.3.2). Continuous variables were expressed as the mean ± standard deviation, and between-group comparisons were performed using the *t* test or Wilcoxon rank-sum test, as appropriate. Categorical variables were expressed as numbers and percentages, and between-group comparisons were performed using the *χ*^2^ test or Fisher's exact test, as appropriate. Univariable and multivariable logistic regression analyses were used to evaluate factors associated with subsequent PIBO. In the univariable analyses, *ORs* for continuous variables were reported per one-unit increase on their original measurement scales. In the conventional multivariable combined model, continuous predictors were standardized using the training-cohort mean and standard deviation, and the corresponding adjusted *ORs* were interpreted per 1-SD increase. *ORs* for binary variables represented presence vs. absence.

After application of the eligibility criteria, all included children had complete data for the prespecified predictors and outcome. Complete-case analysis was therefore performed, and no missing-value imputation was required. *ORs* were reported with *95% CIs.* AUC values were reported to three decimal places, *ORs* to two decimal places, and exact two-sided *P* values to three decimal places; *P values* <0.001 were reported as *P* <0.001. Statistical significance was defined as a two-sided *P* <0.05.

### 2.7 Reporting framework and model reproducibility

This prediction-model study was reported with reference to the TRIPOD + AI reporting guideline. To facilitate reproducibility, the full model specification, including the intercept, regression coefficients, predictor coding, standardization parameters, and probability-transformation formula, is reported in Section 2.5.

## 3 Results

### 3.1 Comparison of baseline characteristics between the training and validation cohorts

A total of 220 hospitalized children with lower respiratory tract infection who underwent bronchoscopy and BALF testing for clinical indications were included, including 155 in the model-development cohort and 65 in the internal holdout cohort.

During follow-up, 49 children met the diagnostic criteria for PIBO, corresponding to an observed proportion of 22.3% in this selected, higher-risk cohort. PIBO outcome ascertainment was generally completed approximately 2 months after the index infection. Exact patient-level diagnostic intervals were unavailable; therefore, a mean or median time to diagnosis could not be reported. All 49 children who met the diagnostic criteria for PIBO had persistent or recurrent respiratory symptoms, compatible follow-up HRCT findings, and specialist-confirmed clinical assessments. Pulmonary function testing was used as supportive evidence when technically feasible but was not required for outcome classification. The exact number of children with technically acceptable pulmonary function results could not be reliably determined from the retrospective records.

PIBO was diagnosed in 35 of 155 children in the training cohort and 14 of 65 children in the validation cohort, with observed proportions of 22.6% and 21.5%, respectively. The difference between the two cohorts was not statistically significant (*P* = 0.900). The baseline characteristics of the model-development and internal holdout cohorts are summarized in Table 1.

**Table 1:** Baseline characteristics of the model-development and internal holdout cohorts.

Variable	Model development (*N* = 155)	Internal holdout (*N* = 65)	*P* value
Age, months	33.94 ± 17.67	30.71 ± 18.40	0.120
Sex			0.077
Male	94 (61%)	31 (48%)	
Female	61 (39%)	34 (52%)	
Weight, kg	13.13 ± 3.52	13.00 ± 3.92	0.400
Premature birth	26 (17%)	7 (11%)	0.300
Underlying disease	21 (14%)	17 (26%)	0.024
Previous wheezing	52 (34%)	18 (28%)	0.400
Pathogen			0.300
Adenovirus	52 (34%)	26 (40%)	
Mixed infection	30 (19%)	8 (12%)	
Mycoplasma pneumoniae	23 (15%)	13 (20%)	
RSV	36 (23%)	10 (15%)	
Unknown	14 (9.0%)	8 (12%)	
Fever duration, days	8.59 ± 3.13	8.45 ± 2.93	>0.900
Hospital stay, days	12.67 ± 4.67	11.98 ± 4.74	0.400
Oxygen therapy	62 (40%)	27 (42%)	0.800
PICU admission	37 (24%)	18 (28%)	0.600
Mechanical ventilation	29 (19%)	11 (17%)	0.800
WBC, 10^9^/L	10.42 ± 3.35	11.11 ± 3.24	0.130
Neutrophil percentage, %	63.94 ± 11.56	63.52 ± 13.18	0.800
CRP, mg/L	32.98 ± 17.94	27.84 ± 16.64	0.033
PCT, ng/mL	0.48 ± 0.37	0.58 ± 0.43	0.120
LDH, U/L	361.30 ± 79.23	380.74 ± 74.11	0.061
BALF neutrophil percentage, %	52.93 ± 14.81	51.46 ± 15.44	0.300
BALF MMP-9, ng/mL	318.00 ± 125.67	332.87 ± 143.25	0.800
BALF IL-8, pg/mL	319.43 ± 150.22	304.72 ± 123.24	0.700
BALF MPO, ng/mL	249.69 ± 120.76	246.56 ± 103.57	>0.900
PIBO during follow-up	35 (23%)	14 (22%)	0.900

Data are presented as mean ± SD or *n* (%). *P values* were calculated using Wilcoxon rank-sum test, Pearson *χ*^2^ test, or Fisher exact test as appropriate. BALF, bronchoalveolar lavage fluid; PIBO, post-infectious bronchiolitis obliterans. The proportions of PIBO reflect the observed event proportions in this selected cohort and should not be interpreted as population-based prevalence estimates.

### 3.2 Comparison of clinical characteristics between children with and without PIBO during follow-up in the training cohort

In the training cohort, compared with children who did not develop PIBO during follow-up, those who developed PIBO during follow-up had a higher proportion of adenovirus or mixed infection (*P* < 0.001), a longer duration of fever (10.29 ± 3.26 vs. 8.10 ± 2.93 days, *P* < 0.001), and significantly higher rates of PICU admission (43% vs. 18%, *P* = 0.003) and mechanical ventilation (46% vs. 11%, *P* < 0.001). Regarding laboratory indicators, the PIBO group showed higher neutrophil percentage and lactate dehydrogenase (LDH) levels. For BALF inflammatory biomarkers, the levels of MMP-9, IL-8, and MPO were significantly higher in the PIBO group than in the non-PIBO group, with values of 391.06 ± 144.56 vs. 296.69 ± 111.54 ng/mL, 402.82 ± 197.32 vs. 295.11 ± 124.25 pg/mL, and 334.92 ± 156.52 vs. 224.83 ± 95.46 ng/mL, respectively. All differences were statistically significant (all *P* < 0.001), as shown in Table 2.

**Table 2:** Baseline characteristics according to PIBO status during follow-up.

Variable	No PIBO (*N* = 120)	PIBO (*N* = 35)	*P* value
Age, months	33.43 ± 16.71	35.71 ± 20.82	0.800
Sex			0.600
Male	74 (62%)	20 (57%)	
Female	46 (38%)	15 (43%)	
Weight, kg	13.06 ± 3.45	13.36 ± 3.80	0.800
Premature birth	24 (20%)	2 (5.7%)	0.047
Underlying disease	15 (13%)	6 (17%)	0.600
Previous wheezing	39 (33%)	13 (37%)	0.600
Pathogen			<0.001
Adenovirus	34 (28%)	18 (51%)	
Mixed infection	18 (15%)	12 (34%)	
Mycoplasma pneumoniae	22 (18%)	1 (2.9%)	
RSV	32 (27%)	4 (11%)	
Unknown	14 (12%)	0 (0%)	
Fever duration, days	8.10 ± 2.93	10.29 ± 3.26	<0.001
Hospital stay, days	12.51 ± 4.63	13.23 ± 4.83	0.400
Oxygen therapy	48 (40%)	14 (40%)	>0.900
PICU admission	22 (18%)	15 (43%)	0.003
Mechanical ventilation	13 (11%)	16 (46%)	<0.001
WBC, 10^9^/L	10.35 ± 3.22	10.65 ± 3.81	0.700
Neutrophil percentage, %	62.61 ± 11.31	68.47 ± 11.41	0.006
CRP, mg/L	31.86 ± 17.24	36.84 ± 19.92	0.200
PCT, ng/mL	0.45 ± 0.37	0.57 ± 0.37	0.056
LDH, U/L	347.13 ± 76.10	409.91 ± 70.86	<0.001
BALF neutrophil percentage, %	51.65 ± 14.59	57.32 ± 14.92	0.065
BALF MMP-9, ng/mL	296.69 ± 111.54	391.06 ± 144.56	<0.001
BALF IL-8, pg/mL	295.11 ± 124.25	402.82 ± 197.32	<0.001
BALF MPO, ng/mL	224.83 ± 95.46	334.92 ± 156.52	<0.001

Data are presented as mean ± SD or *n* (%). *P* values were calculated using Wilcoxon rank-sum test, Pearson *χ*^2^ test, or Fisher exact test as appropriate.

BALF, bronchoalveolar lavage fluid; PIBO, post-infectious bronchiolitis obliterans.

### 3.3 Univariable and multivariable logistic regression analyses of factors associated with PIBO during follow-up

In the conventional multivariable logistic regression model, each 1-SD increase in fever duration (*OR* = 1.72, *95% CI,* 1.03–3.01; *P* = 0.045) and BALF MPO level (*OR* = 1.93, *95% CI,* 1.13–3.61; *P* = 0.028), together with adenovirus infection (*OR* = 4.68, *95% CI,* 1.36–20.03; *P* = 0.021) and mechanical ventilation (*OR* = 4.04, *95% CI,* 1.30–13.08; *P* = 0.016), remained associated with subsequent PIBO after mutual adjustment. The complete univariable logistic regression results are presented in Table 3.

**Table 3:** Univariable logistic regression for factors associated with PIBO during follow-up in the training cohort.

Variable	OR (95% CI)	*P* value
Age, months	1.01 (0.99–1.03)	0.500
Female sex	1.21 (0.56–2.58)	0.630
Weight, kg	1.02 (0.92–1.14)	0.661
Premature birth	0.24 (0.04–0.88)	0.063
Underlying disease	1.45 (0.48–3.92)	0.482
Previous wheezing	1.23 (0.55–2.67)	0.609
Adenovirus infection	7.85 (3.08–24.26)	<0.001
Mycoplasma pneumoniae infection	1.18 (0.53–2.57)	0.676
Fever duration, days	1.27 (1.12–1.46)	<0.001
Hospital stay, days	1.03 (0.95–1.12)	0.421
Oxygen therapy	1.00 (0.46–2.14)	1.000
PICU admission	3.34 (1.47–7.57)	0.004
Mechanical ventilation	6.93 (2.90–17.05)	<0.001
WBC, 10^9^/L	1.03 (0.92–1.15)	0.649
Neutrophil percentage, %	1.05 (1.01–1.09)	0.010
CRP, mg/L	1.02 (0.99–1.04)	0.151
PCT, ng/mL	2.26 (0.85–5.98)	0.096
LDH, U/L	1.01 (1.01–1.02)	<0.001
BALF neutrophil percentage, %	1.03 (1.00–1.06)	0.048
BALF MMP-9, ng/mL	1.01 (1.00–1.01)	<0.001
BALF IL-8, pg/mL	1.00 (1.00–1.01)	0.001
BALF MPO, ng/mL	1.01 (1.00–1.01)	<0.001

ORs for continuous variables represent the effect per unit increase. BALF, bronchoalveolar lavage fluid; OR, odds ratio; CI, confidence interval.

Because BALF biomarkers were measured during the acute hospitalization, the observed associations may reflect both the intensity of local airway inflammation and the severity of the initial infection. Among the three BALF biomarkers evaluated, only MPO remained associated with subsequent PIBO after adjustment for age, fever duration, adenovirus infection, mechanical ventilation, PICU admission, MMP-9, and IL-8. Nevertheless, this adjusted association should not be interpreted as evidence that MPO is a PIBO-specific, causal, or independently validated prognostic biomarker. The present analysis was designed to estimate associations and predictive performance rather than to evaluate causal biological pathways.

### 3.4 Development of prediction models and evaluation of overall performance

Analysis of the conventional logistic regression models showed that the clinical, BALF biomarker, and combined models yielded AUCs of 0.846 (*95% CI*, 0.780–0.913), 0.815 (*95% CI*, 0.736–0.893), and 0.860 (*95% CI,* 0.795–0.926), respectively, in the training cohort. In the internal holdout cohort, the corresponding AUCs were 0.718 (*95% CI*, 0.584–0.853), 0.703 (95% CI, 0.560–0.846), and 0.723 (*95% CI,* 0.587–0.859), respectively.

Although the conventional combined model had a slightly higher AUC than the clinical model in the training cohort (0.860 vs. 0.846), its AUC was also only slightly higher in the internal holdout cohort (0.723 vs. 0.718). The clinical relevance of this small numerical difference was uncertain and was further examined using bootstrap validation and ridge-penalized sensitivity analysis.

Calibration analysis showed no strong evidence of lack of fit in the training cohort, with a Hosmer–Lemeshow test *P* value of 0.507. However, the corresponding *P* value was 0.016 in the internal holdout cohort, indicating suboptimal calibration. Risk-stratification analysis showed that the observed PIBO event proportions in the model-defined high- and low-risk groups were 43.2% and 3.7% in the training cohort and 36.0% and 12.5% in the internal holdout cohort, respectively. Overall, the combined model showed relatively favorable apparent discrimination in the training cohort but only moderate discrimination and suboptimal calibration in the internal holdout cohort. Although adding BALF biomarkers produced small numerical changes in discrimination, clear incremental predictive value beyond the clinical model was not demonstrated. Therefore, the combined model should be considered exploratory and requires external multicenter validation and potential recalibration before clinical application.

### 3.5 ROC curves and discriminative performance of the prediction models

In the training cohort, the conventional logistic clinical, BALF biomarker, and combined models yielded AUCs of 0.846, 0.815, and 0.860, respectively. In the internal holdout cohort, the corresponding AUCs were 0.718, 0.703, and 0.723, indicating moderate discrimination. Although the combined model had the highest AUC in both cohorts, its numerical advantage over the clinical model was small, particularly in the internal holdout cohort (0.723 vs. 0.718). Therefore, the conventional logistic analyses alone did not demonstrate clinically meaningful incremental discrimination from adding BALF biomarkers. The ROC curves are shown in Figure 1.

**Figure 1:**
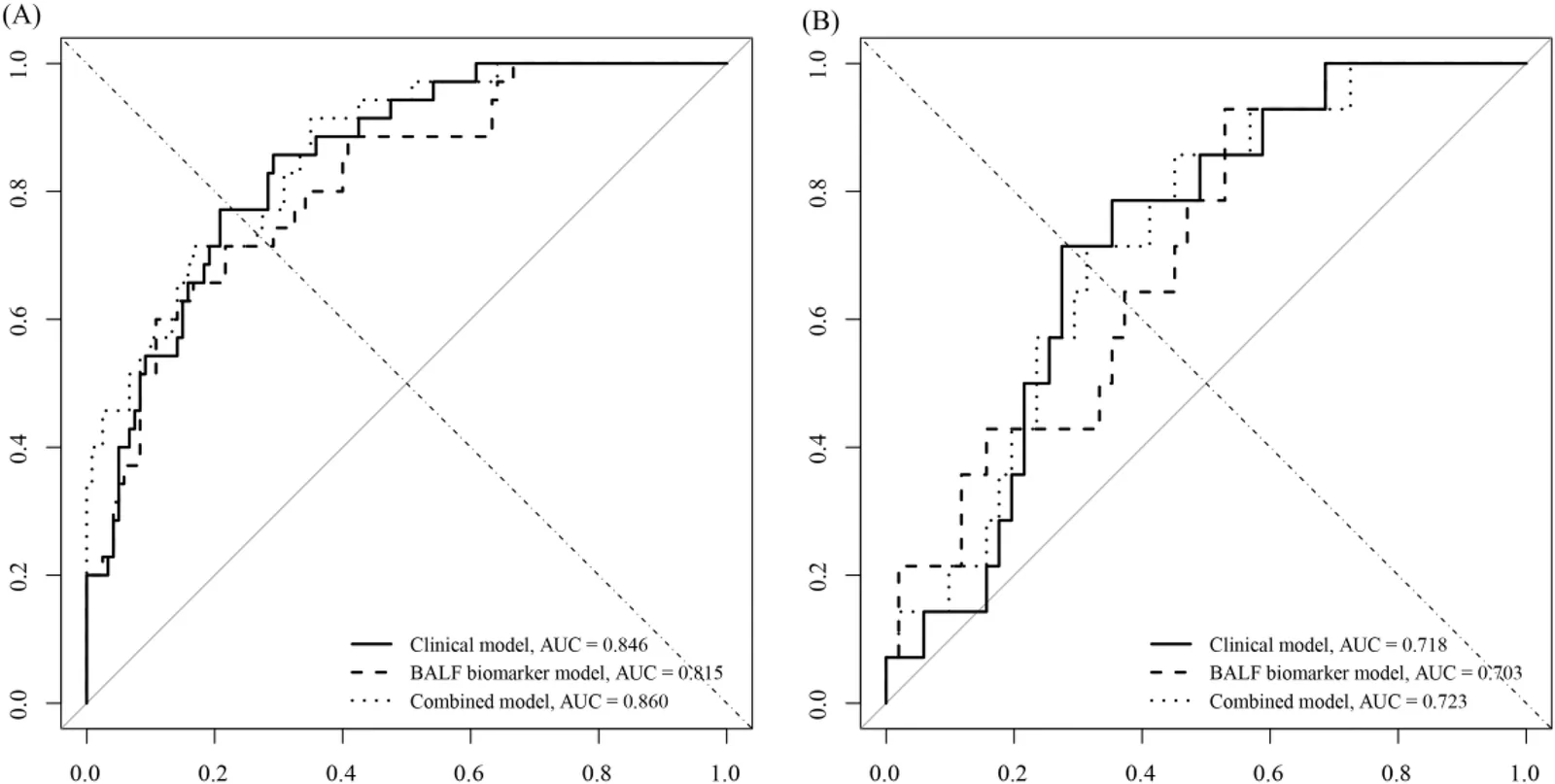
Receiver operating characteristic curves of the clinical model, BALF biomarker model, and combined model. **(A)** ROC curves in the training set. **(B)** ROC curves in the internal holdout cohort. BALF, bronchoalveolar lavage fluid; ROC, receiver operating characteristic; AUC, area under the curve.

At the cutoff of 0.151 derived from the training cohort, the sensitivity of the combined model decreased from 91.4% in the training cohort to 64.3% in the validation cohort, whereas specificity remained similar at 65.0% and 68.6%, respectively. Accuracy was 71.0% in the training cohort and 67.7% in the validation cohort. The reduction in sensitivity, together with the suboptimal calibration observed in the validation cohort (Hosmer–Lemeshow test, *P* = 0.016), indicates limited performance stability in the internal holdout validation cohort and supports the need for external validation and possible recalibration before clinical application.

### 3.6 Calibration curve analysis of the combined model

The calibration curve showed that the predicted probabilities of the combined model were generally consistent with the observed outcome proportions in the model-development cohort. The Hosmer–Lemeshow test showed no evidence of poor fit (*P* = 0.507). However, in the internal holdout cohort, the Hosmer–Lemeshow test yielded a *P* value of 0.016, indicating some evidence of calibration deviation. Therefore, further optimization through expansion of the sample size or model recalibration is still required. The calibration curves are shown in Figure 2.

**Figure 2:**
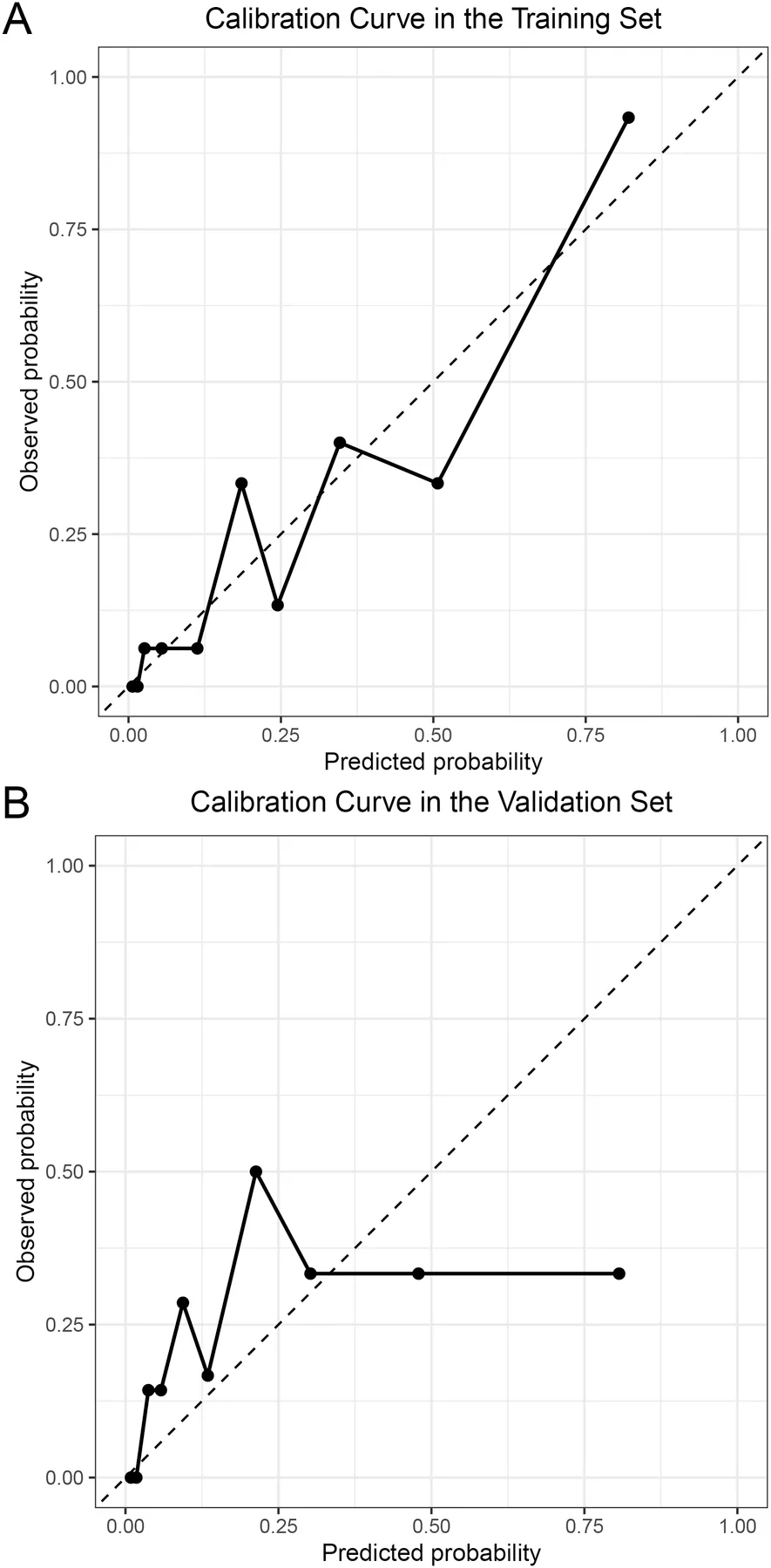
Calibration curves of the combined model. **(A)** Calibration curve in the training set. **(B)** Calibration curve in the internal holdout cohort. The dashed line represents perfect calibration, and the solid line represents the observed calibration performance.

### 3.7 Decision curve analysis of the prediction models

Decision curve analysis in the training cohort showed that the combined model provided greater apparent net benefit than the treat-all and treat-none strategies across parts of the evaluated threshold range. However, this analysis was limited to the training cohort. Because the internal holdout analyses did not demonstrate clear incremental predictive value of the combined model over the clinical model, the DCA findings should be interpreted as exploratory and do not establish incremental clinical utility of BALF biomarkers. The DCA curves are shown in Figure 3.

**Figure 3:**
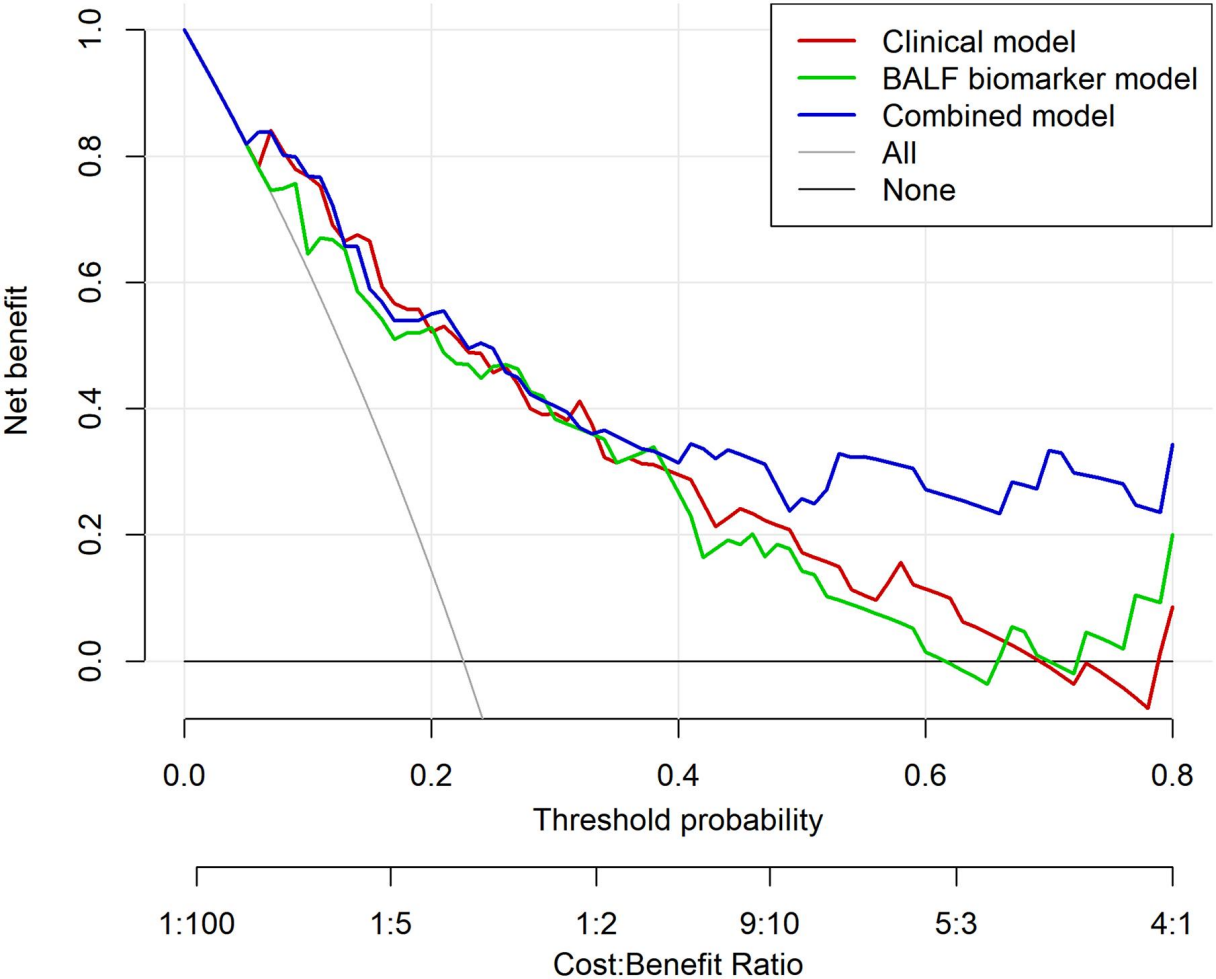
Exploratory decision curve analysis of the prediction models in the training cohort.

The curves show the apparent net benefit of the clinical, BALF biomarker, and combined models in the training cohort. Because these findings were not evaluated and confirmed in an independent external cohort, they should not be interpreted as evidence of established clinical utility.

### 3.8 Nomogram derived from the conventional combined model

The nomogram was constructed directly from the full conventional combined logistic regression model and incorporated age, fever duration, adenovirus infection, mechanical ventilation, PICU admission, and BALF levels of MMP-9, IL-8, and MPO. It therefore used the same eight predictors, coding scheme, standardization procedure, and regression coefficients as the conventional combined model reported in Table 4 and the model equation. The resulting nomogram is shown in Figure 4.

**Table 4:** Multivariable logistic regression model for PIBO during follow-up in the training cohort.

Variable	Adjusted OR (95% CI)	*P* value
Age, per SD	1.26 (0.78–2.05)	0.344
Fever duration, per SD	1.72 (1.03–3.01)	0.045
Adenovirus infection	4.68 (1.36–20.03)	0.021
Mechanical ventilation	4.04 (1.30–13.08)	0.016
PICU admission	0.94 (0.30–2.80)	0.910
BALF MMP-9, per SD	1.50 (0.88–2.72)	0.157
BALF IL-8, per SD	1.35 (0.81–2.44)	0.286
BALF MPO, per SD	1.93 (1.13–3.61)	0.028

Continuous predictors were standardized using the training-cohort means and standard deviations; adjusted *ORs* therefore represent the association per 1-SD increase. Binary predictors were coded as 1 for presence and 0 for absence. The estimates represent mutually adjusted associations and should not be interpreted as causal effects or independently validated predictive effects.

**Figure 4:**
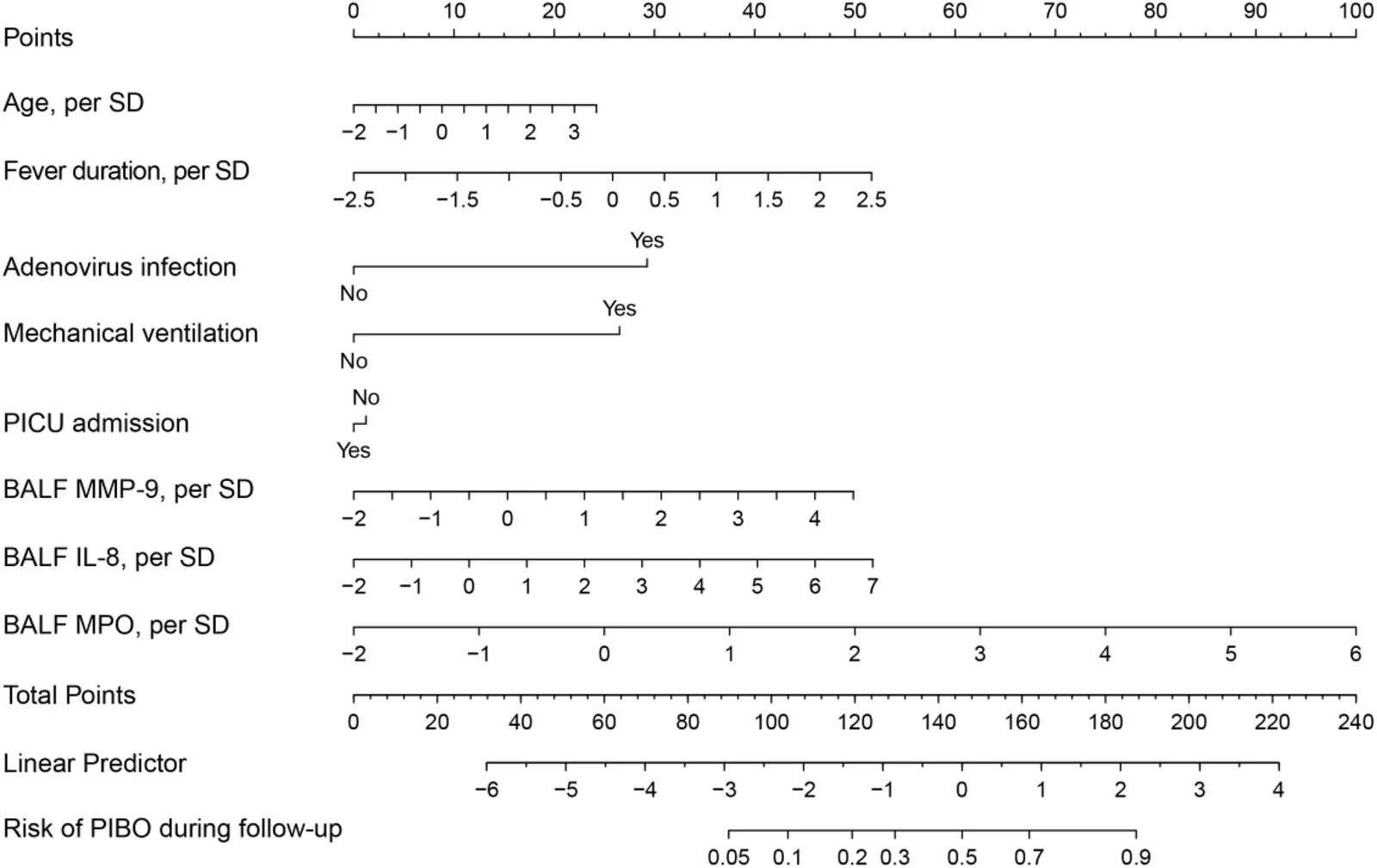
Nomogram derived from the conventional combined logistic regression model for estimating the risk of PIBO during follow-up.

Age, PICU admission, BALF MMP-9, and BALF IL-8 did not individually reach statistical significance in the multivariable analysis. However, they were retained because the combined model was prespecified to evaluate the joint contribution of five clinical predictors and three BALF biomarkers. Predictor retention was not based solely on individual *P* values, and no *post hoc* backward elimination was performed. The nomogram should be interpreted as a graphical representation of the exploratory full model rather than as a model containing only independently significant factors.

Because the effective sample size was limited and internal holdout performance was moderate, the nomogram requires external multicenter validation and should not currently be used for clinical decision-making.

### 3.9 Assessment of overfitting and penalized regression sensitivity analysis

The training cohort contained 35 PIBO events. The clinical, BALF biomarker, and combined models included 5, 3, and 8 predictor parameters, corresponding to EPV values of 7.0, 11.7, and 4.4, respectively. The low EPV of the combined model indicated a risk of overfitting. Detailed results for discrimination, classification performance, calibration, risk stratification, bootstrap internal validation, and ridge-penalized sensitivity analysis are presented sequentially in Tables 5–10.

**Table 5:** Discriminative performance of the prediction models.

Dataset	Model	AUC *(95% CI)*
Training	Clinical model	0.846 (0.780–0.913)
Training	BALF biomarker model	0.815 (0.736–0.893)
Training	Combined model	0.860 (0.795–0.926)
Internal holdout	Clinical model	0.718 (0.584–0.853)
Internal holdout	BALF biomarker model	0.703 (0.560–0.846)
Internal holdout	Combined model	0.723 (0.587–0.859)

AUC, area under the receiver operating characteristic curve; CI, confidence interval.

**Table 6:** Classification performance of the combined model at the training-set-derived cutoff.

Dataset	Cutoff	Sensitivity	Specificity	PPV	NPV	LR+	LR−	Accuracy
Training	0.151	91.40%	65.00%	43.20%	96.30%	2.61	0.13	71.00%
Internal holdout	0.151	64.30%	68.60%	36.00%	87.50%	2.05	0.52	67.70%

The cutoff was determined in the training set using the Youden index and then applied to the internal holdout cohort. PPV, positive predictive value; NPV, negative predictive value. LR+, positive likelihood ratio; LR−, negative likelihood ratio. Likelihood ratios were calculated from sensitivity and specificity at the cutoff derived from the training cohort.

**Table 7:** Calibration assessment of the combined model.

Dataset	Hosmer-Lemeshow *χ*^2^	df	*P* value
Training	7.278	8	0.507
Internal holdout	18.733	8	0.016

A non-significant Hosmer-Lemeshow test suggests no strong evidence of lack of fit. The significant result in the internal holdout cohort indicates that calibration may require improvement.

**Table 8:** Risk stratification based on the combined model.

Dataset	Risk group	*N*	Events	Event rate
Training	High risk	74	32	43.2%
Training	Low risk	81	3	3.7%
Internal holdout	High risk	25	9	36.0%
Internal holdout	Low risk	40	5	12.5%

Risk groups were defined using the optimal cutoff derived from the training set.

**Table 9:** Bootstrap internal validation of the conventional combined model.

Performance measure	Apparent estimate	Optimism	Optimism-corrected estimate
AUC	0.86	0.038	0.822
Calibration slope	1	0.226	0.774
Calibration intercept	0	0.184	−0.184
Brier score	0.113	−0.021	0.134

The combined model included eight predictor parameters and was developed using 35 PIBO events. Optimism was estimated using 1,000 bootstrap resamples. For the Brier score, lower values indicate better overall predictive accuracy.

**Table 10:** Ridge-penalized logistic regression sensitivity analysis.

Dataset	Model	AUC (*95%CI*)
Training	Clinical model	0.822 (0.747–0.896)
Training	BALF biomarker model	0.815 (0.738–0.893)
Training	Combined model	0.848 (0.780–0.917)
Internal holdout	Clinical model	0.706 (0.570–0.842)
Internal holdout	BALF biomarker model	0.703 (0.560–0.846)
Internal holdout	Combined model	0.742 (0.608–0.876)

The difference between the ridge combined and clinical models in the internal holdout cohort was not statistically significant (paired DeLong test, *P* = 0.475).

In 1,000-resample bootstrap internal validation, the apparent AUC of the conventional combined model was 0.860, and the optimism-corrected AUC was 0.822. The optimism-corrected calibration slope was 0.774, the calibration intercept was −0.184, and the Brier score was 0.134. These findings indicated moderate optimism and some degree of coefficient overfitting in the apparent training performance.

Ridge-penalized logistic regression was performed as a sensitivity analysis. The ridge combined model yielded AUCs of 0.848 in the training cohort and 0.742 in the internal holdout cohort. In the internal holdout cohort, the ridge combined model had a numerically higher AUC than the ridge clinical model (0.742 vs. 0.706); however, the difference was not statistically significant (paired DeLong test, *P* = 0.475). Therefore, although ridge penalization produced a modest numerical improvement in the holdout AUC of the combined model, it did not establish clear incremental discrimination from adding BALF biomarkers. The incremental predictive contribution of BALF biomarkers was additionally explored in the internal holdout cohort. The continuous NRI for the combined model compared with the clinical model was 0.151 (95% bootstrap CI, −0.434 to 0.737), and the IDI was 0.032 (95% bootstrap CI, −0.025 to 0.096). Both confidence intervals included zero, indicating that these exploratory measures did not demonstrate a clear improvement in risk classification or discrimination after adding BALF biomarkers.

## 4 Discussion

In this selected cohort, BALF biomarkers were measured during the index hospitalization, whereas PIBO was generally ascertained at the follow-up assessment approximately 2 months after the index infection. This temporal sequence allowed these biomarkers to be evaluated as baseline candidate prognostic factors but did not establish that they represented an early biological stage of PIBO. Higher acute-phase BALF MPO levels remained associated with subsequent PIBO after adjustment for selected clinical severity indicators; however, this association should be distinguished from incremental predictive performance. The conventional combined model yielded only a minimal numerical increase in holdout AUC compared with the clinical model (0.723 vs. 0.718). Similarly, in the ridge sensitivity analysis, the combined model had a higher holdout AUC than the clinical model (0.742 vs. 0.706), but the difference was not statistically significant. Therefore, the incremental prognostic contribution of BALF biomarkers remains uncertain.

The inclusion of a predictor in a multivariable prediction model should be distinguished from statistical significance in an individual coefficient test. Age, PICU admission, BALF MMP-9, and BALF IL-8 did not individually reach statistical significance, but they were retained to preserve the prespecified full model and the intended comparison between the clinical and combined models. Removing variables *post hoc* according to *P* values could lead to unstable selection and additional optimism, particularly given the limited number of PIBO events. Nevertheless, neither the AUC comparison nor the exploratory NRI and IDI analyses demonstrated clear incremental predictive value from adding BALF biomarkers.

The clinical findings of the present study largely confirmed previously reported associations. Adenovirus infection, prolonged fever, PICU admission, mechanical ventilation, hypoxemia, and extensive pulmonary involvement have repeatedly been associated with subsequent PIBO in previous cohorts and meta-analyses. Therefore, the associations of adenovirus infection, longer fever duration, and mechanical ventilation observed in our study should be interpreted primarily as confirmatory rather than novel findings. Their consistency with previous evidence supports the clinical plausibility of the present cohort and model.

The present findings should also be interpreted in the context of earlier international studies that established the clinical framework of pediatric PIBO. A prospective HRCT cohort described mosaic attenuation, air trapping, bronchiectatic changes, and other structural abnormalities as characteristic imaging manifestations of post-infectious bronchiolitis obliterans in children (13). In a large case-control study, adenovirus infection and the requirement for mechanical ventilation were identified as major factors associated with the subsequent development of bronchiolitis obliterans (14). Other historical cohorts reported persistent wheezing, dyspnea, atelectasis, lobar collapse, and bronchiectatic changes after severe lower respiratory tract infection (15). Long-term follow-up has further demonstrated that obstructive pulmonary-function abnormalities may persist throughout childhood despite variable clinical trajectories (16). These established observations support the clinical plausibility of the associations identified in the present cohort but also indicate that the clinical predictors evaluated here should be regarded primarily as confirmatory rather than novel.

The principal exploratory contribution of this study was the evaluation of acute-phase BALF inflammatory biomarkers measured during the index hospitalization. Among MMP-9, IL-8, and MPO, only higher BALF MPO levels remained associated with subsequent PIBO after adjustment for selected clinical severity indicators and the other BALF biomarkers. Nevertheless, this finding should be interpreted cautiously, because MPO may reflect the intensity of neutrophilic inflammation and the severity of the initial infectious insult rather than a PIBO-specific biological process. Accordingly, BALF MPO should be regarded as a hypothesis-generating candidate prognostic marker rather than an established clinically useful biomarker.

Elevated BALF MPO, MMP-9, and IL-8 levels were compatible with a more pronounced local inflammatory state during severe lower respiratory tract infection. However, the present observational study did not assess serial biomarker trajectories, airway histopathology, cellular sources, or causal biological pathways. In addition, the timing of bronchoscopy and treatment exposure before BALF collection was not standardized. Therefore, the observed biomarker concentrations should be interpreted as correlates of the acute inflammatory and clinical state rather than evidence that MPO, MMP-9, or IL-8 directly causes airway remodeling or subsequent PIBO. Whether these biomarkers participate in biological pathways leading to chronic small-airway injury cannot be determined from the present data. Prospective longitudinal and mechanistic studies are required to clarify these relationships.

This study has several limitations. First, the number of PIBO events was limited. The combined model included eight predictor parameters but was developed using only 35 PIBO events in the training cohort, corresponding to an EPV of 4.4. This low EPV indicated a substantial risk of coefficient instability and overfitting. In bootstrap internal validation, the AUC decreased from an apparent value of 0.860 to an optimism-corrected value of 0.822, and the corrected calibration slope was 0.774, confirming some degree of overfitting. Ridge penalization was used as a sensitivity analysis and produced a validation AUC of 0.742 for the combined model; however, this was not significantly higher than that of the clinical model. Although bootstrap validation and penalization helped quantify and reduce model optimism, they could not fully compensate for the limited effective sample size. Moreover, the internal holdout cohort contained only 14 PIBO events, resulting in imprecise validation estimates. Therefore, the model should be considered exploratory and requires validation in a larger independent cohort. Second, all participants were hospitalized children who underwent bronchoscopy and BALF testing for clinical indications. This selection process likely enriched the cohort for children with more severe, persistent, or diagnostically challenging lower respiratory tract infections and introduced selection bias. Therefore, the observed proportion of 22.3% should not be interpreted as the prevalence of PIBO among all children with lower respiratory tract infection. The findings and model performance are primarily applicable to similar hospitalized children undergoing clinically indicated bronchoscopy and may not be generalizable to children with mild infections, children managed in outpatient settings, or those who do not undergo bronchoscopy. Third, BALF inflammatory biomarkers were measured at a single time point during the acute hospitalization, whereas PIBO was generally ascertained approximately 2 months after the index infection. In addition, bronchoscopy and BALF collection were not performed at a uniform protocol-defined time point. Bronchoscopy was generally undertaken when clinically significant pulmonary consolidation was identified and when the expected diagnostic or therapeutic benefit was considered to outweigh the procedural risk. Because the timing depended on the individual disease course, imaging findings, response to treatment, and clinical assessment, BALF samples may have represented different stages of the acute inflammatory response. This temporal heterogeneity, together with treatment exposure before bronchoscopy, may have introduced measurement variability and residual confounding. Consequently, the temporal trajectory of MMP-9, IL-8, and MPO from acute infection to chronic small-airway disease could not be evaluated. Elevated biomarker levels may have reflected the severity of the initial infection, local neutrophilic inflammation, treatment exposure, or the timing of bronchoscopy rather than a PIBO-specific process. Although the multivariable model adjusted for selected severity indicators, including fever duration, PICU admission, and mechanical ventilation, residual confounding by unmeasured or incompletely measured disease severity remains possible. Moreover, the absence of improved validation performance after adding BALF biomarkers limits conclusions regarding their incremental prognostic value. Prospective studies with standardized and serial BALF or peripheral biomarker measurements are required to clarify their temporal and independent prognostic roles. The moderate validation AUCs and the absence of improved discrimination after adding BALF biomarkers limit the clinical relevance of the combined model. Therefore, the current findings do not support performing bronchoscopy or BALF biomarker testing solely for PIBO risk prediction. Another limitation is the absence of chest CT-based predictors from the nomogram. Chest CT is an important component of clinical assessment in children with suspected PIBO. In this study, follow-up HRCT findings were used to ascertain the outcome and could not be included as predictors without introducing information leakage. However, standardized acute-phase CT features were also unavailable, which reduced the clinical completeness of the model and may have omitted relevant prognostic information regarding the extent and pattern of pulmonary involvement. Consequently, the nomogram is not intended to replace imaging assessment. Future prospective studies should incorporate prespecified acute-phase CT variables or radiomic features and evaluate whether they improve prediction beyond clinical and BALF variables. Fourth, although the combined model performed well in the training cohort, calibration deviation was observed in the validation cohort, suggesting that further recalibration and external validation are required before direct clinical application. PIBO outcome ascertainment was generally performed approximately 2 months after the index infection, but the exact diagnostic timing was not standardized and could not be reconstructed at the individual-patient level. Differences in follow-up scheduling, disease evolution, and the timing at which persistent symptoms and characteristic HRCT abnormalities became apparent may therefore have influenced outcome ascertainment. Because exact patient-level dates were unavailable, the mean or median interval from infection onset to fulfillment of the PIBO diagnostic criteria could not be calculated. Future prospective studies should use predefined assessment time points, prospectively record the date on which the diagnostic criteria are first fulfilled, and consider time-to-event methods when diagnostic timing varies. The interval from infection to PIBO diagnosis was not included as a predictor because it was a post-baseline variable available only after outcome occurrence and was undefined in children without PIBO. Nevertheless, the absence of standardized diagnostic timing remains a limitation. Future prospective studies should use predefined assessment time points or time-to-event methods to account for variation in diagnostic timing. Pulmonary function testing was not consistently available because of the young age and limited cooperation of some children. Consequently, PIBO outcome ascertainment relied primarily on persistent clinical manifestations, compatible follow-up HRCT findings, exclusion of alternative diagnoses, and specialist assessment. Although this approach reflects real-world pediatric practice, incomplete pulmonary function data may have introduced some uncertainty in outcome classification. Future studies should conduct multicenter prospective cohort studies with larger sample sizes and include children with different pathogens, disease severities, and follow-up durations. In addition, dynamic BALF biomarkers, peripheral blood inflammatory markers, radiomic features, pulmonary function parameters, and long-term prognostic data should be integrated to further optimize prognostic models for subsequent PIBO and to evaluate their potential value in determining follow-up frequency, guiding timely intervention, and stratifying prognosis. Complete historical information regarding replicate measurements, assay-specific quality-control parameters, and formal laboratory blinding to subsequent PIBO outcome status could not be reliably reconstructed from the retrospective records. Moreover, medication exposure before BALF collection was neither standardized according to a study protocol nor comprehensively modeled, and antibiotics, systemic corticosteroids, antiviral agents, or other anti-inflammatory therapies may have influenced BALF inflammatory biomarker concentrations. These factors may have introduced measurement variability and residual confounding.

In this selected hospital-based cohort, the associations of adenovirus infection, prolonged fever, and mechanical ventilation with subsequent PIBO were consistent with previous evidence. Higher acute-phase BALF MPO levels remained associated with subsequent PIBO after adjustment for selected clinical factors and the other BALF biomarkers. Although adding BALF biomarkers produced small numerical changes in discrimination and reclassification measures, clear incremental predictive value beyond the clinical model was not demonstrated. The nomogram represents an exploratory full model rather than a tool containing only statistically significant predictors. The model and the proposed prognostic contribution of BALF MPO require prospective external validation and potential recalibration in larger multicenter cohorts before clinical application.

## Data Availability

The raw data supporting the conclusions of this article will be made available by the authors, without undue reservation.
